# Supplementary material for: Are carbon emissions trading and green financial instruments synergistic? -Comprehensive quantitative research based on content analysis
Source: PLoS One. 2024 Mar 7;19(3):e0298601. doi: 10.1371/journal.pone.0298601 (PMC10919601; doi:10.1371/journal.pone.0298601)
Supplement: S1 Data — (ZIP) [file pone.0298601.s001.zip › Data/地级市Z_F工作报告.zip!/╔╜╢1⁄2/║╩╘≤╩╨/└┤╫╘╣1⁄2╓┌║┼í╛┬φ┐╦╩2╛▌═°í┐_2011.docx]

各位代表:

　　现在，我代表市人民政府，向大会作政府工作报告，请予审议，并请市政协委员和其他列席会议的同志提出意见。

　　一、2010年及“十一五”时期工作回顾

　　过去的一年，面对极为复杂的国内外经济环境，全市人民在市委的坚强领导下，深入贯彻科学发展观，认真落实省委、省政府决策部署，求真务实，埋头苦干，促进了经济社会健康快速发展。预计实现地区生产总值1145亿元，比上年增长15%左右，高于全省3个百分点；完成地方财政收入84.6亿元，增长39.8%，增幅居全省第一位；城市居民人均可支配收入14419元，增长13.2%；农民人均纯收入5655元，增长12%；金融机构存贷款余额分别达到1092.6亿元和803.5亿元，分别比年初增加192.6亿元和172.9亿元，增幅均居全省第二位，圆满完成了市十七届人大四次会议确定的各项任务。一年来，主要做了以下工作：

　　（一）着力开展项目建设推进年、提升年活动，招商引资和大项目建设实现新突破。规模以上固定资产投资完成582亿元，增长22.6%，其中亿元以上在建项目完成320亿元，增长26%。新落地招商引资亿元以上项目230个，总投资930亿元。东明石化120万吨高硫重油综合利用、玉皇化工10万吨顺丁橡胶，郓城洪达焦化，曹县凯雷圣奥（一期），成武达驰电气750千伏级变压器，单县舜亦新能源（一期）、宇泰光电，市开发区玉皇化工20万吨苯乙烯等185个过亿元项目相继竣工投产；牡丹区华润电厂、华盛荣镁合金，郓城赵楼煤矸石电厂，市开发区聚隆能源，巨野铁雄新沙20万吨甲醇，成武呈祥风力发电塔，东明方明化工聚合切片等260个过亿元项目开工建设，为经济发展提供了强劲动力。

　　（二）大力推进“1655”企业成长计划，工业化进程进一步加快。全市规模以上工业企业达到2001家，新增222家；完成主营业务收入2436亿元、利税295亿元，分别增长28%和62%。规模以上工业增加值占地区生产总值的比重达到51.1%，提高0.6个百分点。“1655”企业完成主营业务收入1400亿元，增长40%，占规模以上工业的58.3%。主营业务收入过10亿元的达到22家，过亿元的632家，分别增加6家和223家。郓城富仕达食品、成武九州科技、曹县富洋木业、巨野龙泰果蔬等4家企业成功上市，募集资金6.3亿元。

　　（三）深入实施城建三年纲要，城镇化建设迈出重大步伐。全市城镇化率达到35%，提高2个百分点。市区、县城控制性详细规划实现全覆盖，县域村镇体系规划、乡镇总体规划全部完成。开工基础设施建设工程335个，其中市区71个；完成拆迁面积386万平方米，开工房地产面积1650万平方米，竣工1100万平方米，其中市区开工680万平方米，竣工405万平方米。曹州牡丹园升级改造、赵王河下游和洙水河公园景观建设基本竣工，中山路、长城路等43条道路新修、改造工程完工，新烈士陵园、市开发区污水处理厂建成启用。市区安装路灯3355盏，栽植乔灌木30多万棵，绿化覆盖率达到40.3%。菏泽大剧院获得全国建筑工程质量最高奖——鲁班奖，赵王河公园景观工程获得全国市政工程质量最高奖——市政金杯奖，并入选国家水利风景区。县城和小城镇建设全面加快。全市启动整村建设项目298个，开工建设10万户，改造危房4.5万户。集中开展了城乡环境综合整治、公路迎国检、规范治理户外广告等活动，城乡面貌明显改观。

　　（四）加快推进农业产业化，农村经济全面发展。农业综合生产能力不断提高。粮食总产113亿斤，实现连续七年增产；棉花总产474万担，瓜菜总产188亿斤；新增造林面积23万亩；新建林下规模养殖场区249个、大棚1803个；水产品总产13万吨。农业产业化进程进一步加快。新增规模以上农业产业化龙头企业50家、农民专业合作组织731个、优质农产品基地40万亩，我市被批准为“全国牡丹高新技术产业基地”，佳农果蔬晋升为国家级农业产业化龙头企业；新增无公害农产品、绿色食品、有机食品认证58个。农业生产生活条件得到较大改善。疏浚中小河道和田间沟渠8896公里，引送黄河水11.5亿立方米。启动新农村建设310个，涌现出了东明玉皇新村等一批先进典型。

　　（五）强化科技创新、节能减排和土地集约节约利用，转变发展方式取得明显成效。全市取得重要科技成果236项，其中16项被列入省重点推广计划，14项获得省以上奖励。实施技改项目482项，完成投资210亿元，增长45.3%。市级以上高新技术企业发展到131家。全市高新技术产业完成产值640亿元，增长43%；占规模以上工业的25.7%，提高3.4个百分点。我市被确定为“国家知识产权试点城市”,单县被确定为“国家级绿色能源示范县”和“山东省高技术产业基地”。完成节能技改项目125项，淘汰高耗能设备892台套，能耗水平明显下降。新建改造污水管网311公里，重点排污企业“十个一”工程全面竣工，化学需氧量、二氧化硫排放量分别下降1.4%和0.6%。开发整理新增耕地9400亩，盘活闲置低效用地1.1万亩，为保障大项目建设提供了条件。

　　（六）狠抓“四大载体”建设，服务业实现快速发展。重点城区、重点园区、重点企业、重点项目完成服务业投资142亿元，增长77.5%。全市服务业增加值实现333亿元，增长16.5%。实现社会消费品零售总额657亿元，增长19%；各类专业批发市场交易额724亿元，增长28%。主营业务收入3000万元以上的工业企业全部实现了主辅业分离，新增地方税收1.2亿元。完成进出口总额18.1亿美元，增长44.2%，其中出口12.1亿美元，增长39.2%。我市被确定为“中国木草柳出口基地”。全年接待国内外游客650万人次，增长25%，实现旅游总收入36亿元，增长32.4%。鄄城孙膑旅游城被评为国家4A级旅游景区。

　　（七）积极办好十件民生实事，社会更加和谐稳定。新增城镇就业9.2万人、社会保险参保49.7万人次，牡丹区、市开发区新农保试点全面实施，东明县正式启动。城乡低保标准有了较大提高，新建保障性住房5570套。新建改造中小学校舍57.9万平方米，城乡教师工资实现县域内统一标准发放，公开招聘教师1431名。公共卫生服务项目顺利实施，单县基本药物制度试点和巨野、郓城、东明3县扩面全面启动，新农合参合率达到99.9%。新建乡镇综合文化站37个、农村文化大院1015家、农家书屋1000家，送戏送电影下乡计划超额完成，元代沉船考古发掘产生了重大影响。市区新增集中供热面积220万平方米、天然气用户1.1万户，新开通公交线路8条、新上公交车100辆；改造农村公路1062公里，新建户用沼气池2.4万个，新增自来水受益人口60万人。产品质量和食品药品安全专项整治活动扎实有效。安全生产和社会治安形势持续稳定。监察、审计、统计、人口计生、广播电视、人民武装、外事侨务、民族宗教、防震减灾、红十字、气象、档案、史志、体育、人防、老龄、残联等各项事业都取得了新的成绩。

　　各位代表，2010年的发展为全面完成“十一五”奋斗目标做出了重大贡献，提供了坚实保障。纵观“十一五”，面对复杂多变的严峻形势，我们攻坚克难，开拓进取，走过了极不平凡的历程。五年来，我们始终把实现更好更快发展作为第一要务，抢抓“突破菏泽”战略机遇，团结一致，同心同德，大干苦干，有效克服了国际金融危机影响，圆满完成了“十一五”既定目标，全市经济社会发展步入了历史上最好最快的时期。

——综合实力大幅攀升。全市地区生产总值由483亿元增加到1145亿元，年均增长15.8%。地方财政收入由30亿元增加到84.6亿元，年均增长32%，总量在全省前移3个位次，上升到第12位。金融机构各项存款余额由498.3亿元增加到1092.6亿元，年均增长22.8%；各项贷款余额由398.3亿元增加到803.5亿元，年均增长18.8%。城市居民人均可支配收入由7342元增加到14419元，年均增长13.5%；农民人均纯收入由3092元增加到5655元，年均增长12.8%。

——产业结构不断优化。三次产业比由32:43.6:24.4调整到19.2:51.7:29.1，二三产业比重提高12.8个百分点。规模以上工业企业个数由821家发展到2001家，增加1180家；主营业务收入由427亿元增加到2436亿元，年均增长41%；利税由29亿元增加到295亿元，年均增长59%。高新技术产业产值占比由15.2%增加到25.7%，提高10.5个百分点。新能源、新材料、新医药产业实现了快速发展。

——固定资产投资稳步增长。全社会固定资产投资由257亿元增加到582亿元，年均增长22.6%。东明石化、步长制药、洪业化工、玉皇化工、达驰电气、尚舜化工等一批骨干企业加速膨胀，铁雄新沙、巨润玻璃、艺达家纺、金正大国际等一批重点项目相继竣工投产，彭庄、龙堌、赵楼、郭屯四对矿井先后投产运行，郓城矿、万福矿建设和陈蛮庄矿、张集矿前期工作进展顺利，曹县煤田勘探进入详查阶段，经济发展后劲显著增强。

——城镇化步伐明显加快。全市城镇化率达到35%，提高8.5个百分点。有30余项城乡规划获省部级以上奖励。市区累计完成投资320亿元，大剧院、演武楼、赵王河公园、长江路黄河路改造等一批重点工程完工，市政基础设施逐步配套完善，城市综合开发顺利推进，创建成中国优秀旅游城市和省级园林城市。县城建设快速推进，小城镇开发全面启动。市、县经济开发区承载功能不断提高。东明至巨野、曹县至鄄城一级公路基本建成，洙水河航道菏泽段工程完工，日东输油管道建设顺利推进。日南高速、济菏高速、德商高速（南段）相继建成，支撑经济发展的能力进一步增强。

　　——节能减排取得重大进展。万元GDP能耗累计降低22%，化学需氧量、二氧化硫排放量分别下降15%和8%，圆满完成了省下达的节能减排目标，生态市建设成效明显。

　　——社会事业全面进步。全部免除了城乡义务教育阶段学生学杂费和农村中小学生课本费。新型农村合作医疗制度实现全覆盖，标准不断提高。列入国家级、省级非物质文化遗产名录分别达到30项和60项。成功承办了十一届全运会武术散打决赛。累计新增城镇就业46万人。先后启动实施了城镇居民基本医疗保险、生育保险和新型农村社会养老保险，各类社会保险新增参保229万人次。城乡低保和大病救助标准不断提高。累计建成保障性住房17922套。对口支援北川灾后重建工作提前一年完成。

　　各位代表，事实充分说明，过去的五年，是我们积极应对挑战、抢抓机遇，乘势而上、大步跨越的五年；是整体实力明显增强，转方式调结构取得重大进展的五年；是基础设施日益完善，城乡面貌发生明显变化的五年；是社会事业全面进步，人民群众得到更多实惠的五年；也是我市在省内、国内地位和影响不断提升的五年。

　　过去五年的成功实践，为“十二五”及今后长远发展奠定了坚实的基础，积累了宝贵的经验。一是必须立足菏泽实际创新工作思路。思路决定出路，创新推动发展。市委市政府确定的实现“四个发展”，建设“四大基地一大产业”，加快推进工业化、城镇化、农业产业化和繁荣发展服务业等工作思路，既符合科学发展观要求，更符合菏泽实际，也是实现更好更快发展的必然选择，必须一以贯之地坚持好、落实好。二是必须坚持不懈地抓好大项目建设。大项目建设是经济工作的总抓手、生命线和首要任务。充分利用资源优势和产业基础，加大招商引资和大项目建设力度，加快骨干企业培育，是扩大经济总量、调整优化结构、改变欠发达面貌的根本途径。不论遇到什么情况，都必须坚定不移、持之以恒地抓下去，这样才能实现后来居上、跨越发展。三是必须不断创新完善工作机制。良好的工作机制是推动“四个发展”的重要保障。几年来，我们建立了分工负责、督查调度、大项目季度观摩、考核奖惩等保障和促进发展的工作机制，推行了服务承诺、限时办结和部门主要负责人电话公开三项制度，有力地促进了干部作风转变，保证了各项决策部署的贯彻落实。四是必须始终坚持执政为民。一切权力属于人民，执政为民是各级政府的崇高使命。必须牢记全心全意为人民服务的宗旨，进一步实现好、维护好、发展好最广大人民的根本利益。更加注重保障和改善民生，让人民群众共享改革发展的成果。坚持一切为了人民，一切依靠人民，这是我们事业发展的源泉和力量所在。五是必须形成干事创业的强大合力。人心齐，泰山移。风正才能气顺，心齐才能劲足。只有团结协作，步调一致，同心、同向、同力，我们的事业才能成功，前途才会更加辉煌。

　　各位代表，过去五年的成绩来之不易。这是市委统揽全局、正确领导的结果，是市人大、政协和社会各界有效监督、大力支持的结果，是全市干部群众拼搏奉献、扎实苦干的结果。在此，我代表市人民政府，向全市广大干部群众，向各民主党派、工商联、人民团体和社会各界人士，向驻菏部队、武警官兵和中央、省属单位，向所有关心支持菏泽发展的朋友们，表示崇高的敬意和衷心的感谢!

　　成绩令人鼓舞，但发展中仍存在一些不容忽视的矛盾和问题。主要是：经济总量小，财政收入少，人均占有水平低；产业结构不合理，大企业大项目不够多，自主创新能力弱；城镇化水平低，服务业发展滞后；节能减排压力加大，资源环境约束强化；政府职能转变还不到位，社会管理能力和公共服务水平还比较低，政府工作人员的服务质量和效率有待进一步提高，等等。对此，我们一定高度重视，认真加以解决。

　　二、“十二五”时期目标任务和总体要求

　　“十二五”时期，是我市工业化、城镇化的加速推进期，是经济社会发展的创新转型期，也是乘势而上、加快赶超的关键期，更是打造鲁苏豫皖四省交界地区科学发展高地的攻坚期。我们既面临诸多风险挑战，更面临难得的发展机遇。全市上下加快发展的决心更大、劲头更足、作风更实，“十二五”发展的动力更强、环境更优、条件更好。因此，我们必须更加珍惜机遇、抓住机遇、用好机遇，为实现“十二五”更好更快发展勤奋工作、努力奋斗，孜孜以求、开拓奋进。

　　全市经济社会发展的总体要求是：以邓小平理论和“三个代表”重要思想为指导，认真贯彻落实科学发展观，紧紧围绕科学发展这个主题，牢牢把握转方式、调结构、增效益、上水平这条主线，以改善民生为根本出发点，认真实施工业化和城镇化双轮驱动战略，扎实推进“五大基地一大产业”建设，努力推动全市经济社会科学发展、和谐发展、跨越发展、更好更快发展，在打造鲁苏豫皖交界地区科学发展高地征程上迈出坚实步伐。

　　“十二五”时期主要发展目标是:

　　——经济实力显著增强。到2015年，地区生产总值达到2500亿元，年均增长14%。地方财政收入达到200亿元，年均增长20%。经济总量达到山东省西部地区平均水平，人均地区生产总值接近全国平均水平。

　　——产业结构进一步优化。三次产业比调整为10:55:35，二三产业比重提高9.2个百分点。“五大基地一大产业”基本建成。

　　——城乡建设协调发展。全市城镇人口达到440万人左右，其中市区人口100万人以上，城镇化率45%以上。城区基础设施配套和新农村建设达到全省平均水平。

　　——生态优势更加明显。完成省下达的节能减排约束性目标，森林覆盖率达到35%，生态环境进入良性循环，人居环境显著改善，生态菏泽、绿色菏泽的新形象进一步展现。

　　——社会建设全面加强。教育质量不断提升，城乡居民医疗保健水平进一步提高，公共文化服务体系基本形成。社会管理制度趋于完善，社会更加和谐稳定。

　　——生活水平明显提高。全市城镇年均新增就业9万人，转移农村劳动力14万人。城乡最低生活保障标准翻一番，城镇基本养老、基本医疗、失业保险覆盖率分别达到95%、95%和85%，新型农村养老保险实现全覆盖。城市居民人均可支配收入、农民人均纯收入年均分别增长12%以上。

　　工作中将重点把握好以下几个方面:

　　一是坚持结构调整，加快推进发展方式转变。坚定不移地推进工业化、城镇化、农业现代化和服务业发展。把扩大优质增量作为转方式、调结构的关键措施，狠抓招商引资和大项目建设，不断膨胀经济总量，壮大经济实力，在发展中促转变，在转变中谋发展。

　　二是坚持自主创新，着力营造产业竞争优势。建立完善创新体系，强化产学研合作，壮大创新人才队伍，提高自主创新能力，推动传统产业转型升级，促进战略性新兴产业加快发展，在新一轮发展中抢占先机、争得主动，实现创新驱动、内生增长的可持续发展。

　　三是坚持统筹兼顾，积极促进城乡一体发展。大力实施新型城镇化战略，加快构建以中心城市为龙头，县城和重点乡镇为主体，基层社区为基础的现代城镇体系，推动城乡规划、基础设施建设、公共服务一体化，形成城镇体系完善、产业布局合理、城乡发展协调、人口加快聚集的发展格局。

　　四是坚持绿色增长，大力加强生态文明建设。推进节能减排，强化污染防治，节约集约利用土地，积极发展循环经济、绿色经济、低碳经济，加快建设资源节约型、环境友好型社会，全力打造经济发展新优势。

　　五是坚持改革开放，不断增强发展动力和活力。继续深化政府机构、财税、投资、农村综合配套改革，健全完善有利于科学发展的体制机制。深入实施对外开放战略，加大招商引资力度，转变外贸发展方式，全力提升外向型经济的质量和水平。

　　六是坚持民生优先，共建共享幸福和谐社会。强化执政为民理念，每年为群众办一批实事好事，促进社会事业全面发展和人民生活水平不断提高。

　　各位代表，未来五年是菏泽充满希望的五年，也是大有作为的五年。尽管前进道路上还会遇到一些困难和挑战，只要我们振奋精神、抢抓机遇，发挥优势、扎实苦干，就一定能够圆满完成“十二五”奋斗目标，力促经济发展再创新优势，人民生活再上新水平，整体实力再上新台阶。

　　三、2011年主要任务和工作重点

　　2011年是“十二五”开局之年，做好今年的工作对于“十二五”开好局、起好步至关重要。今年经济社会发展的主要预期目标是：地区生产总值增长14%以上；地方财政收入增长16%以上；规模以上工业增加值增长19%以上；千万元以上项目固定资产投资增长20%以上；社会消费品零售总额增长19%以上；外贸进出口增长15%以上；外商直接投资增长15%以上；城市居民人均可支配收入和农民人均纯收入均增长12%以上；人口自然增长率控制在8.4‰以内；节能减排完成削减任务。

　　为实现上述目标，将着重做好以下八个方面的工作：

　　（一）全力提升大项目建设的质量和水平，努力实现转方式调结构的新跨越

　　突出转方式调结构这条主线，必须靠优质增量做支撑，靠高质量、高水平的大项目来实现。今年必须把提升大项目建设的质量和水平，作为经济工作的重中之重，作为加快转方式调结构、提升产业和区域竞争力的关键措施，推动项目建设尽快由“多、快”向“多、快、大、好”转变。确保全年新开工、续建亿元以上大项目300个以上，完成投资400亿元以上，其中竣工投产100个以上。

　　集中精力招大引强。加强对世界500强、国内500强等大企业、大财团发展方向、投资重点的跟踪分析研究，搞好对接合作，加快引进一批龙头型、基地型、旗舰型项目。每个县区力争引进5个投资10亿元以上、10个投资亿元以上的大项目。研究制定具体考核办法，明确和落实市级领导以及市直相关部门的招商目标任务。按照国家产业政策和行业标准，严把新上项目质量关，低水平的项目一律拒之门外，坚决避免质量差的项目占用宝贵的生产要素和资源。进一步完善大项目考核办法，对投资10亿元以上项目及高新技术项目、产业链高端项目、利用境外资金项目，观摩评比和年终考核时增加相应分值，以此引导投向，优化投资结构。

　　加大项目推进力度。进一步健全推进机制，认真落实包保责任制，全力加快在建项目建设，确保郓城煤矿、华润电厂（一期）、日东输油管道、玉皇化工8万吨稀土顺丁橡胶、巨润玻璃（二期）、铁雄新沙（二期）、富海化工、鑫泽铜业等100个大项目投产达效。扎实推进万福煤矿、张集煤矿、陈蛮庄煤矿、东明石化300万吨重质油综合利用、玉皇化工20万吨丙烯和碳酸二甲酯等100个续建大项目建设，确保如期竣工，尽早发挥效益。抓紧完善东药药业22.5万吨丁辛醇、洪达化工15万吨己二酸等大项目报批手续，确保新开工亿元以上项目100个以上。

　　提高项目策划水平。围绕我市“五大基地一大产业”建设，精心策划编制一批产业链延伸项目、战略性新兴产业项目、高新技术产业项目、传统产业升级改造项目以及其他财源项目，确保储备亿元以上生产性项目投资达到300亿元以上。

　　加快园区经济转型升级。加强园区软硬件配套建设，着力提高园区承载能力。积极引导重点产业、重点企业、重点项目向园区集中，推动园区产业向集约化、专业化和高端化发展。清理整合园区内不符合产业定位、没有效益的项目和企业，腾出有限的资源向大项目、好项目倾斜。加快推进市开发区和高新区建设，进一步扩大面积，完善配套，吸引更多的生产要素加快向市区转移，着力提高市区首位度。以省级工业园区为依托，高标准制定产业集群发展规划，大力培育新能源、新材料、生物医药、煤化工、石油天然气、精细化工、机械电子、纺织服装、食品、林木等十大产业集群，力争主营业务收入占规模以上工业的88%以上。加大战略性新兴产业培育力度，新能源、新材料、新医药产业主营业务收入超过500亿元，增长35%以上。立足煤炭、石油等资源优势，以建设绿色、生态化工基地为方向，着力打造西起东明、东至巨野的重化工产业隆起带，进一步推动产业集群集聚发展。

　　（二）全力推进“1655”企业成长计划，努力培育转方式调结构的主力军

　　转方式调结构，关键在企业，重点在企业，希望也在企业。“1655”企业是我市工业的主导力量，是振兴菏泽的希望所在，必须坚持政银企联手，配套联动，着力培育真正立市立县的百亿强企。确保 “1655”企业实现主营业务收入2100亿元，增长50%以上；新增主营业务收入过10亿元的企业5家以上。

　　加大调度督导力度。严格落实目标责任制，将计划目标层层分解到县区、企业。充分发挥工业运行指挥部的作用，坚持定期调度和现场办公制度，强化运行分析，完善应急预案，搞好煤电油运等生产要素协调供给，确保企业满负荷生产，实现最大效益。

　　企业要立大志、干大事、创大业。企业家是企业发展的灵魂，有大企业家才能有大企业。充分发挥李湘平、王金书、余庆明、马效坤、徐承秋、赵步长等优秀企业家的模范带头作用，引导企业家提升境界，拓宽视野，增强干事创业的责任感、紧迫感，以大手笔策划论证大项目、好项目，以项目建设支撑企业做大做强。要大力开展对外重组和经济技术合作，加速膨胀步伐。要利用先进技术、工艺、设备，加快提升现有装备水平和产品档次。全年完成技改投资260亿元，增长30%。要全面加强企业管理，切实抓好职工队伍建设，大力提升企业综合素质和核心竞争力。要充分认识上市对于规范企业管理、解决融资难题、优化资本结构、提升企业品牌和形象的重要意义，积极做好企业上市工作，新增上市企业3家以上。

　　强化政策扶持和服务。继续落实好扶持中小企业发展的一系列政策措施，进一步研究制定加快“1655”企业发展的意见，在立项、财税、科技、土地、能评、环评、安评等方面加大扶持力度。加快实施质量竞争力提升工程，努力培育一批知名品牌和高端创新产品生产基地，力争新增省级以上名牌产品10个。建立健全激励机制，对贡献突出的优秀企业家进行大张旗鼓地表彰奖励。继续优化金融生态环境，深入推进银企对接，积极搭建融资平台，力争新增贷款150亿元以上。努力拓宽融资渠道，争取曹县等3县区村镇银行开业，2家股份制商业银行在我市设立分支机构，新增各类担保公司20家以上。

　　（三）全力加快改革开放、科技创新步伐，努力为转方式调结构提供不竭动力和强大支撑

以更大的决心和勇气推进改革开放，以更大的力度和气魄提升自主创新能力，尽快推动我市驶入创新驱动、内生增长的发展道路。

　　加快科技进步和创新。继续推进技术创新提升工程行动计划，重点实施一批科技重大专项，争取省级以上重点项目达到35项，实施重大专项10项。着力加强产学研合作，积极培育创新型试点企业，新增市级以上创新型企业10家、技术中心20家。认真落实支持科技创新的政策措施，充分发挥好政府专项资金的作用，引导全社会加大科技投入，确保研发投入达到10亿元，占地区生产总值的0.8%以上。抓好高新技术重点项目建设，扶持高新技术企业加速发展，力争高新技术产业产值完成800亿元。大力实施“人才强市”战略，全市引进博士45人、硕士360人、职业经理人45人以上。

　　进一步深化改革扩大开放。稳步推进基层医疗卫生机构改革，切实抓好基本医疗保障制度建设。深化投资体制改革，促进民间投资快速增长，严格管理政府投资行为。加快县乡兽医体制改革和集体林权制度配套改革。引导和支持企业创建自主品牌，巩固扩大欧美传统市场，开拓东盟、中东、南美等新兴市场，进出口总额完成20亿美元，外商直接投资达到1.3亿美元。

　　（四）全力推进服务业大发展，努力培植转方式调结构的新亮点

　　服务业是我市转方式、调结构的重点和潜力所在。以“四大载体”建设为抓手，进一步推动服务业加快发展，力争服务业增加值占地区生产总值的比重提高1—2个百分点。

　　繁荣发展商贸流通业。加快在建重点商贸流通项目建设，确保菏泽国贸中心、名嘉广场、单县义乌商品城、巨野大元国际商贸城等一批项目按时竣工投运，尽快发挥效益。认真落实国家扩大消费的一系列政策措施，深入实施“万村千乡市场工程”，积极搞好“农超对接”，大力发展新型物流业态，加快传统商贸业优化升级。全市交易额过30亿元的市场达到9处，实现专业批发市场交易额800亿元，增长18%。进一步整顿和规范市场秩序，严厉打击假冒伪劣等商业欺诈行为，切实让人民群众安全消费、放心消费。

　　大力发展现代物流业。以打造区域性商贸物流基地为目标，搞好牡丹医药、东明石化、巨野煤化工等十大物流园区配套建设，力争营业额过100亿元的达到3个。规划建设省级煤炭储配基地和郓城煤化工物流园区。积极扶持交通集团、华瑞、三信等物流企业快速发展，尽快培育一批现代物流企业集团。进一步完善企业二三产业分离后续工作。鼓励发展电子商务、金融服务等新兴服务业。

　　加快发展文化旅游业。以打造“一城四乡一生态”旅游品牌为重点，着力抓好单县浮龙湖生态旅游区、巨野麒麟文化产业园等一批项目建设。组织开展好“好客山东贺年会”活动，精心筹备好国际牡丹花会。力争全年接待国内外游客750万人次，实现旅游总收入46亿元，分别增长22.5%和28%。

　　（五）全力做好节能减排、节约集约用地工作，努力为转方式调结构提供更大空间

　　建设资源节约型、环境友好型社会是加快转变发展方式的重要着力点。我市正处于工业化、城市化快速发展阶段，节能减排任务繁重。必须强化政治责任意识，严格落实目标责任制，确保全面完成节能减排目标。

　　切实抓好节能降耗。严格执行国家能评标准，严控高耗能项目进入。进一步加强重点企业节能监管，深入开展能效对标活动，夯实企业节能管理基础。着力加快重点节能项目建设，大力推广使用节能新产品新技术，抓好高耗能设备的更新改造和淘汰，培植一批循环经济示范工程和示范项目。综合推进建筑节能、公共机构节能、交通节能。

　　扎实推进治污减排。认真落实建设项目环评和“三同时”制度，从源头上控制新污染源产生。在抓好管理减排和结构减排的基础上，重点推进工程减排，加快各县区工业污水处理厂建设，强化污水处理厂和中水回用工程运行监管，加大污水管网建设力度，新铺设污水管网150公里，确保在迎接国家淮河流域水污染防治考核中取得好成绩。完善治理空气质量联动机制，进一步加强对燃煤锅炉、汽车尾气、城区烧烤等污染源的治理，争取空气良好率明显提升。

　　坚持节约集约利用土地。坚决执行最严格的耕地保护制度，加强执法监察长效机制建设，严肃查处各类违法违规用地行为。充分利用挖潜整合、盘活存量、增减挂钩等多种途径，努力拓宽用地空间，为经济社会发展提供保障。

　　（六）全力加快现代农业发展步伐，努力为转方式调结构奠定坚实基础

　　发展现代农业是繁荣农村经济、促进农民增收的重要举措，是转方式调结构的重要内容。必须以发展现代农业为突破口，推动农业和农村经济健康发展。

　　积极推进农业结构调整。努力稳定粮食面积，提高单产，力争总产达到113亿斤。认真实施蔬菜、渔业、畜牧、果业、苗木花卉五大产业振兴规划，加快牡丹、中药材和山药等特色产业发展，经济作物面积达到750万亩，新增45万亩。突出抓好美国白蛾、禽流感等动植物疫病防控，确保不发生大的灾害。

　　加快发展农业产业化经营。继续扶持52家支撑企业、30家龙头企业膨胀发展，新增规模以上农业产业化龙头企业50家以上、农民专业合作组织300家以上。加强农产品质量安全示范区建设，新增标准化生产基地40万亩以上，无公害农产品、绿色食品、有机食品认证40个以上。继续办好中国林产品交易会和农资交易会。

　　努力提高农业科技水平。深入实施科技项目示范和科技特派员工程，全年下派科技特派员1200人，组建利益共同体550个，培训农民30万人次。不断加强实用人才队伍建设，集成推广粮食增产、畜牧良种和防疫等新技术，科技进步对农业经济增长的贡献率达到50%以上。

　　大力改善农业生产条件。着力推进土地综合整治治理，建设高标准基本农田30万亩以上。积极推广农机规模化作业，机械化水平达到80%以上。加快万福河二期工程进度，启动建设巨野大野水库，争取开工建设定陶刘楼水库、成武九女湖水库和鄄城雷泽水库，继续实施闫潭、谢寨、苏泗庄等灌区治理，认真做好8个小农水重点县项目建设，组织开展好农田水利基本建设会战。

　　（七）全力加快城乡一体化建设，努力打造转方式调结构的大平台

　　城镇化是工业化的重要载体，是扩大居民消费的重要切入点，是推动转方式调结构的大平台。全面落实城镇建设三年纲要，加快推进城乡一体化进程，城镇化率达到36.5%以上。

　　高标准搞好市区规划建设管理。编制完成一批重点片区、地段详细规划和专项规划，搞好城市设计和重点工程规划设计。把人防、消防建设纳入城市总体规划。加快赵王河上游治理和下游湿地公园建设，开工建设图书馆、规划展览馆、胜利公园，启动雷泽湖、环城公园二期综合治理，加快博物馆、艺术馆、科技馆建设前期工作步伐，努力打造城市新亮点。打通和提升改造太原路、丹阳东路、长城路等主次干道。深入实施碧水工程，对赵王河引水线路及沿岸进行全线截污，确保水质达到四类标准以上。全面推进数字化城管建设，继续搞好绿化、亮化、美化，综合治理市容市貌，进一步提升城市品位和形象。

　　加快县城、村镇规划建设步伐。加强基础设施建设，大手笔开发建设一批精品工程，不断完善城镇功能。确保各县垃圾处理厂全面竣工投用。加快户籍管理等配套制度改革，鼓励农村人口向城镇有序转移。切实抓好农村住房建设和危房改造，采取合村并居改造、压煤村庄搬迁和农村资源整合等多种方式，启动整村建设300个，新建农村住房6万户，改造危房3万户。

　　积极推进房地产综合开发。进一步加大项目策划、招商和建设力度，全市房地产新开工面积1500万平方米，竣工800万平方米，其中市区新开工550万平方米，竣工300万平方米。

　　加强重大基础设施建设。继续推进德商高速北段、鄄城黄河公路大桥等重点工程进度，力争洙水河航道年底通航，尽快开工建设东新高速、丰东路济宁至定陶段、菏泽火车站改扩建工程。抓紧做好菏鱼高速、济祁高速、东濮高速、郓城新河航道、万福河航道等项目前期工作。坚持把水利作为基础设施建设的优先领域，加快水系生态建设，严格水资源管理，促进水利可持续发展。抓住农网改造机遇，完善电网结构，确保220千伏曹县苏集、110千伏牡丹区秦桥等5项输变电工程建成投用。

　　（八）全力保障和改善民生，努力夯实转方式调结构的着力点和落脚点

　　保障和改善民生是转方式调结构的内在要求，是发展经济的最终目的，必须始终作为政府工作的一项重大使命，紧紧抓在手上。今年集中力量办好十件实事：（1）劳动就业。新增城镇就业9万人以上，转移农村劳动力18万人以上。（2）社会保障。各项社会保险新增扩面25万人次，征缴保险费27亿元；城乡低保标准分别提高到每人每月230元和每人每年1400元；城乡大病救助标准分别提高到年人均4500元和3500元；新建保障性住房6285套。（3）基础教育。新建改造中小学校舍35万平方米以上，新建城区中小学校20所，新增幼儿园200所，公开招录教师1000名以上。（4）公共文化。开工建设牡丹区、曹县等7县区文化馆、图书馆，新建农村文化大院300个、农家书屋2000家；送戏下乡1000场、送电影下乡4万场以上，有线电视数字化平移10万户。（5）医疗卫生。完成市中医院、市精神病防治中心主体工程和市120急救指挥中心建设；启动建设鄄城、东明、单县等县级医院，完成其他县级医院标准化建设；国家基本药物制度覆盖到所有政府办乡镇卫生院和社区卫生服务机构。（6）计划生育。大力实行计划生育利益导向机制，切实稳定低生育水平。及时足额兑现计划生育家庭奖励资金6000万元以上，为农村所有独生子女和合法生育双女绝育家庭免费办理新农合，救助计划生育贫困家庭大学生1000名以上。（7）改善城乡生活条件。市区新增集中供热面积300万平方米、民用天然气用户1万户、公交线路6条；新建改造农村公路600公里、窄危桥30座、户用沼气池1万座，新增农村安全饮水人口30万人。（8）食品药品监管。建立全市统一的食品药品检验中心和药械不良反应监测中心，实施食品药品放心工程和食品安全“百千万”示范工程，确保药品评价性抽验合格率95%以上，餐饮服务群众满意率80%以上。（9）安全生产。创建危险化学品三级以上标准化企业198家、市级安全社区85个；抓好重点行业和领域的安全监管，亿元GDP生产安全事故死亡人数下降到0.2以下。（10）社会稳定。积极开展社会矛盾纠纷排查化解，预防和妥善处置群体性事件。加强基层群防群治组织建设，完善治安防控体系，深入开展严打整治斗争，始终保持严厉打击刑事犯罪高压态势，切实增强人民群众的安全感，确保社会长治久安。抓好突发事件应急管理，提高预防预警和应急处置能力。

　　四、切实加强政府自身建设

　　以转变政府职能、提高行政效能和服务水平为重点，严格按照“四高”要求，进一步加强和改进政府自身建设，全面提高公信力、执行力和创造力，努力建设为民、务实、勤政、廉洁的政府。

　　（一）严格依法行政，强化法治建设。自觉接受市人大及其常委会的法律监督、工作监督和市政协的民主监督，重视群众和舆论监督，广泛听取各民主党派、人民团体的意见和建议。加强法治政府建设，把依法行政贯穿于政府工作的全过程，全面提高依法行政水平。积极推进科学民主决策，规范决策程序，健全重大决策实施效果评估制度，切实强化决策责任。严格规范执法行为，落实执法责任，加强执法监督，做到严格执法、公正执法、文明执法。

　　（二）深入学习研究，提高工作水平。不断增强学习意识，大力弘扬理论联系实际的良好学风，扎实推进学习型政府建设。深入开展调查研究，科学研判发展趋势，准确把握社情民意，积极破解发展难题，不断提高驾驭市场经济、处理复杂问题和推动“四个发展”的能力。

　　（三）加强督导考核，狠抓工作落实。严格按照行政问责制要求，全面实行目标责任制，对每一个指标、每一项任务都量化分解，到岗到人，定进度、定时限、定奖惩。健全各项工作制度，强化调度考核，严格奖勤罚懒，最大限度地催生动力，激发活力，努力营造干事创业的浓厚氛围，真正把心思用在推动发展上，把抓落实体现在成效上，把工作成效体现在民生上。

　　（四）不断改善服务，着力优化环境。加快转变政府职能，积极推进政务公开，加强行政审批中心服务管理。继续推进行政审批制度改革，并对行政审批效能实行实时电子监察。严格实行服务承诺、限时办结、部门主要负责人电话公开三项制度，深入开展民主评议政风行风、企业评议涉企部门等活动，进一步提高工作效率和服务质量。充分发挥纠风办、行政效能投诉中心、市长公开电话受理中心的监督服务职能，进一步改善经济发展环境。

　　（五）强化廉政建设，坚持从严治政。严格执行领导干部《廉政准则》各项规定，认真落实党风廉政建设责任制，重点整治工程建设、涉农资金、土地出让、产权交易等领域的违法违纪行为，坚决纠正损害群众利益的不正之风。大力弘扬节俭创业精神，带头反对铺张浪费，带头抵制拜金主义和奢糜之风，全面树立清正、廉洁的政府形象。

　　各位代表！当前，菏泽经济社会发展已站在新的历史起点上，新形势、新任务对政府工作提出了新要求，全市广大干部群众对政府工作充满了新期待。我们一定在市委的坚强领导下，牢记使命、不负重托，再接再厉、开拓进取，团结一

心、埋头苦干，为全面完成2011年各项任务和“十二五”发展目标而努力奋斗！
